# Anthropometric characteristics of female smallholder farmers of Uganda – Toward design of labor-saving tools

**DOI:** 10.1016/j.apergo.2015.12.010

**Published:** 2016-05

**Authors:** Dana J. Mugisa, Abia Katimbo, John E. Sempiira, William S. Kisaalita

**Affiliations:** a Smallholder Fortunes, P.O. Box 30385, Kampala, Uganda; b College of Agricultural and Environmental Sciences, Makerere University, P.O. Box 7062, Kampala, Uganda; c College of Engineering, University of Georgia, Driftmier Engineering Center, Athens, GA 30602, USA

**Keywords:** Hand-tool design, Mechanization, Anthropometric data, Ethnic difference

## Abstract

Sub-Saharan African women on small-acreage farms carry a disproportionately higher labor burden, which is one of the main reasons they are unable to produce for both home and the market and realize higher incomes. Labor-saving interventions such as hand-tools are needed to save time and/or increase productivity in, for example, land preparation for crop and animal agriculture, post-harvest processing, and meeting daily energy and water needs. Development of such tools requires comprehensive and content-specific anthropometric data or body dimensions and existing databases based on Western women may be less relevant. We conducted measurements on 89 women to provide preliminary results toward answering two questions. First, how well existing databases are applicable in the design of hand-tools for sub-Saharan African women. Second, how universal body dimension predictive models are among ethnic groups. Our results show that, body dimensions between *Bantu* and *Nilotic* ethnolinguistic groups are different and both are different from American women. These results strongly support the need for establishing anthropometric databases for sub-Saharan African women, toward hand-tool design.

## Highlights

• Development of hand-tools to reduce labor burden requires rich anthropometric data.
• Western anthropometric databases do not adequately represent sub-Saharan women.
• Results from measuring 89 women show differences among different groups.
• More measurements are needed for more conclusive results and predictive models.

## Graphical abstract

The plot below reveals general body dimensions’ differences among Ugandan women of *Bantu* (Kiboga, Mbarara and Ngoma locations) and *Nilotic* (Kotido location) ethnolinguistic groups. Principle components (PC1 and PC2) were obtained from analysis of 28 anthropometric measurements from a total of 89 women. The co-clustering of Ngoma and Mbarara women suggest they are identical while the “spread out” along PC2 of the Kiboga women is intriguing and suggests more women should be measured to be able to gain better understanding of the difference there might be between Kiboga women and their other two *Bantu* “sisters”.

**Figure:**
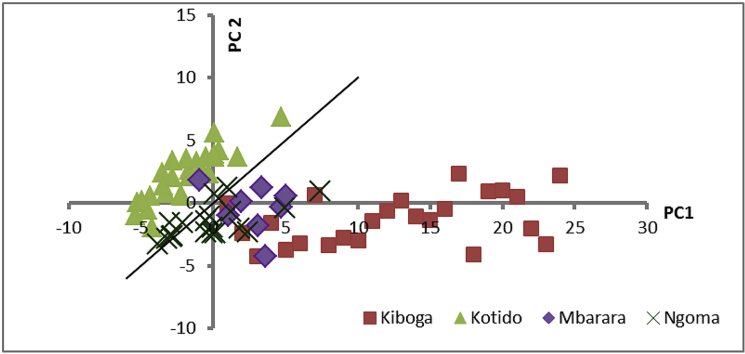

## 1 Introduction

In sub-Saharan Africa, agricultural production predominantly occurs on smallholder farms (Herrero et al., 2010), where women carry a disproportionately higher labor burden (Yisehak, 2008), which is one of the main reasons contributing to the gender asset gap – women are unable to produce for both home and the market (FAO, 2011, Quisumbing et al., 2014). One way to reduce both the labor burden and the gender asset gap is to come up with labor-saving interventions or innovations for these women. In a recent Bill and Melinda Gates Foundation grand challenge exploration, “Labor Saving Strategies and Innovations for Women Smallholder Farmers”, over 50% of the initial funded intervention explorations were hand-tools. This is not surprising; almost a decade ago, a limited range of hand-tools as a productivity problem was identified in a comprehensive FAO report on farm power and mechanization for smallholder farms in sub-Saharan Africa (Sims and Kienzle, 2006).

Optimal design of hand-tools requires applicable comprehensive and context-specific anthropometric or body dimensions and strength data. Although many anthropometric databases are available to inform design, e.g., the repository at the Aerospace Medical Research Laboratories, Dayton, Ohio (USA) and Anthropology Laboratory of Paris University, such data may not be applicable for tool design targeting sub-Saharan women, since the repositories contain data from U.S Army, Air Force personnel, and civilians, or Europeans (Vyavahare and Kallurkar, 2012). However, some data for foreign populations are available in the NASA data bank (Agrawal et al., 2010), but even in this case, sub-Saharan Africans are not well represented.

Most anthropometric measurements made on sub-Saharan Africans have been limited or are completely lacking in parameters that are important for hand-tools design. For example, in one of the studies with a large number of participants, Cheserek et al. (2012) measured six parameters of importance in nutritional status, but no parameters like arm-span or eye-height in sitting and standing positions, that are important in hand-tool design, were measured. In other anthropometric characteristic studies with ethnically different participants, it is evident that there are significant differences in most of the mean dimensions as well as all the body proportions (or prediction equations, e.g., eye height seated from seating height) among the ethnic groups (e.g., Lin et al., 2004, Dewangan et al., 2005, Vyavahare, 2012). It follows that significant differences are likely to be found between the ethnic groups and the American or European databases’ values, lending credibility to the need for large scale anthropometric measurements from sub-Saharan African women for parameters relevant in informing hand-tool design.

Absence of applicable anthropometric measurements may partly be responsible for disappointing results from investments in research and development aimed at producing equipment for smallholder farmers (Sims and Kienzle, 2006). This may also be partly the case for higher incidence of work related injuries such as musculoskeletal disorders among farm workers (Singh and Arora, 2010). However, it should be recognized that, low-adoption rates of hand-tools may also be due to other non-anthropometric or strength factors, such as the tools not fitting well in cultural practices. For example, Muyanja et al. (2009) designed a milk churner for standing operation while women who traditionally churn, do it in a seated posture.

This study was undertaken with the aim of providing preliminary results toward answering two questions. First, how well are existing databases applicable in the design of hand-tools for sub-Saharan African women? Second, given that body dimensions vary according to ethnicity and that sub-Saharan Africans can be grouped on ethnolinguistic basis or as a surrogate for physical characteristic grouping, how different are sub-Saharan women within and across ethnolinguistic lines. To answer these questions, we made anthropometric measurements from women populations from two sub-Saharan ethnolinguistic groups (*Nilotic* and *Bantu*) from the cattle corridor of Uganda.

## 2 Materials and methods

### 2.1 Participants

The study populations were recruited from five locations along the cattle corridor of Uganda namely; Ngoma village/town in Nakaseke district, Nyamilinga and Kabuye villages in Kiboga district, Kanyaryeru village in Kiruhura district and Losilanga village in Kotido district. All procedures performed in this study involving human participants were in accordance with the ethical standards of the University of Georgia Institutional Review Board (STUDY00001461) and the Uganda National Council for Science and Technology (SS 3422). Informed consent was obtained from all individual participants included in this study. We targeted women who were already organized in groups with missions of increasing incomes through ghee-making for home and markets (Katimbo et al., 2015). Our interest in ghee-making was based on the present study objective synergy with a separate objective of the study of working with the same women groups to corroboratively reengineer a labor-saving churner for separating butter-fat from fermented milk (Muyanja et al., 2009, Katimbo et al., 2015). This process precedes ghee-making (Sserunjogi et al., 1998). Three cohorts (Ngoma, Nyamilinga/Kabuye, and Kanyaryeru) were selected from location inhabited by Bahima/Bahororo (*Bantu* ethnicity). One cohort was selected from a location (Kotido) inhabited by Jie (*Nilotic* ethnicity). Ethnicity for each participant was confirmed by directly asking before being accepted as a participant. As is the custom in the cattle corridor, before conducting any measurements, we obtained permission from the local leadership. A token gift of a bar of soap and a kilogram of sugar were provided to each participant.

### 2.2 Body dimensions

**Fig. 1:**
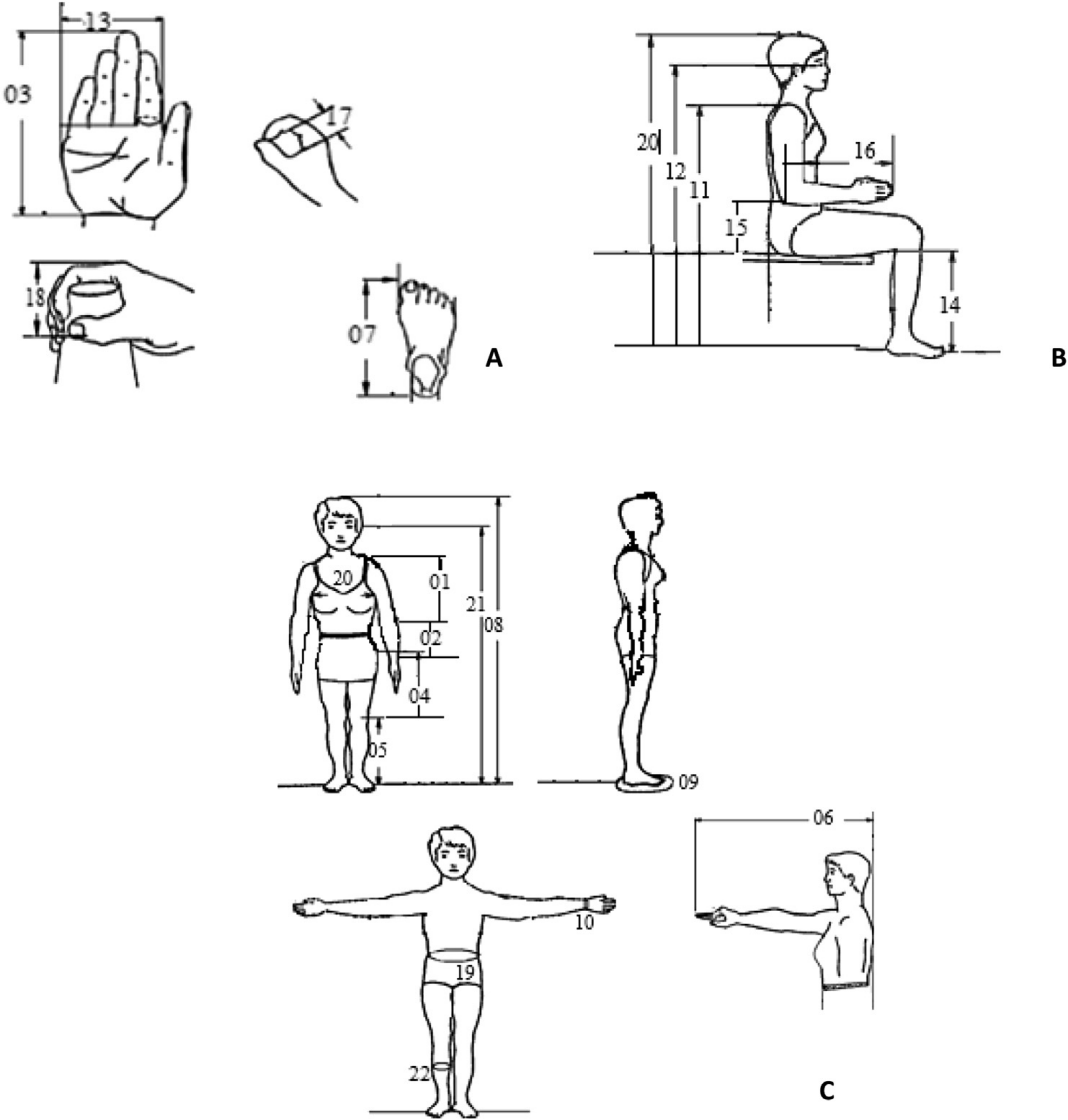
Anthropometric measurements in the standing (A), sitting (B), and siting/standing (C) postures.

The land marks of dimensions measured in standing and sitting postures are shown in Table 1 and Fig. 1. Twenty-eight variables from a list of 37 recommended by Kroemer et al. (1997) were chosen on the basis of relevance to informing hand-tool design and cultural appropriateness from a practical measurement viewpoint. Standard descriptive terminologies (Kroemer et al., 1997) are used. Measurements were conducted following ISO 7250 recommendations (Dewangan et al., 2008). In the standing posture, 15 measurements were made. They included nine lengths form both direct and derived projected heights, five circumferences, and one reach. In the sitting posture, 13 measurements were made. They included seven heights, three breadths, two diameters, and one skinfold thickness.

### 2.3 Equipment and procedures

Body dimensions were measured using equipment from a kit (Rosscraft Centurion Kit, The Human Solution, Austin, TX). A portable weighing scale (0–140 kg, Model S100, Escali, Minneapolis, MN, USA) was used for body weight. A Campbell 20 (54 cm) wide sliding caliper with AP branches was used for measuring foot dimensions. Campbell 10 (18 cm) caliper was used to measure small bone dimensions like breadths of the hands. Segmometer 4 was used to measure height and segmental lengths of different bones. A head square together with a retractable centimeter measuring tape were used to measure both the standing and sitting heights. Slim guide skinfold calipers were used to measure the triceps skinfold thickness. Also anthropometric steel tapes and calipers were used for other length and diameter measurements.

For the standing posture, the participants stood on flat surface (measuring box) with their feet closed, body straight, and the rearmost aspect of the body touching a vertical wall. For the sitting posture, the participants sat with their bodies upright, while the head and shoulders were against a vertical wall. During the measuring process, minimal pressure was exerted when handling the instruments. To achieve a greater uniformity, measurements were taken in accordance with the right hand convention. In addition measurements were mostly collected in the morning hours. The average time taken for measuring one participant was approximately 35 min.

### 2.4 Data analysis

Insights in general differences based on ethnicity and location were gained through Principle Component Analysis (PCA). PCA examines the correlations among a set of variables, in this case measurements, and creates linear combinations of these variables that are uncorrelated with one another. These linear combinations are called principle components (PCs), and usually only a few PCs capture a large portion of the overall variability among the measurements (Hair et al., 1998). The PCs can illustrate differences between subjects. More specific comparative statistical procedures used included ANOVA with tukey adjustment in each case and Student t-test. In all cases differences were considered significant at p-levels of less or equal to 0.05.

## 3 Results and discussions

### 3.1 Principle component analysis

A total of 89 participants were measured and their ethnic breakdown is presented in Table 2. As shown, *Bantu* and *Nilotic* were 64% and 36%, respectively. The *Bantu* were further broken down by three locations of Kiboga (30.3%), Ngoma (23.6%), and Mbarara (10.1%). To first gain an overview in the ways these women differ with respect to ethnicity and location, we used Principle Component Analysis (PCA).

The analysis yielded as many PCs as there were variables (28). There are several ways to determine how many PCs are useful. We chose an approach that relies on a plot of eigenvalues against PC. We visually examined the curve for the “elbow”, where the eigenvalues were no longer greatly changing with increasing PC, which was at the fourth PC. To be conservative, we examined the first six PCs, which explained 73.8% of the variability. We next highlighted the PCs that exhibited correlation greater than 0.5 (strong and positive) or less than −0.5 (strong and negative). Out of the 28 variables 19, 15, and 4 were correlated with PC1, PC2, and PC3, respectively. PC4, PC5, and PC6, were each only correlated with one variable. Thus, we decided to focus on only PC1, PC2 and PC3.

Many measures were significantly correlated with PC1; it may be a measure generally related to size. PC2 was significantly positively related to some of the length and height measurements, but negatively to girth and weight measurements, and so may indicate women who have greater length but less weight. PC3 was strongly significantly related to several seated height measurements. The 4th and 5th were positively and negatively related to hand breadth, respectively. The 6th was highly related to elbow girth.

**Fig. 2:**
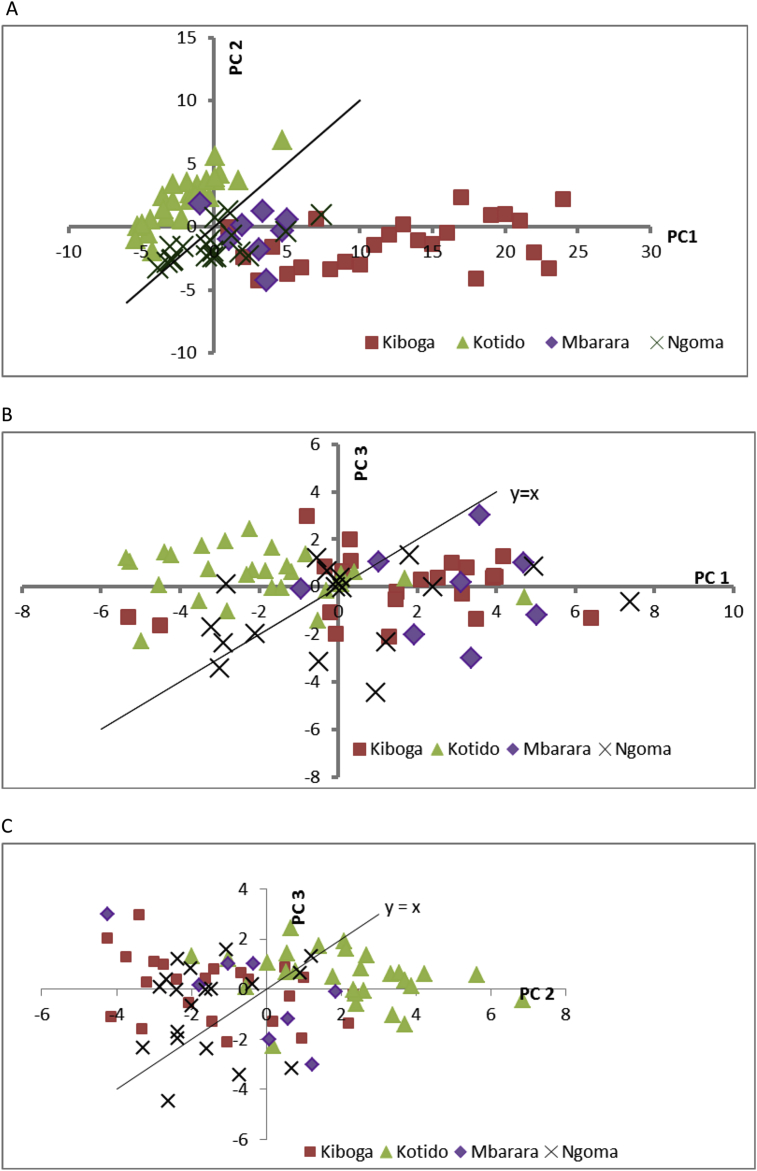
Principle component analysis (PCA) of anthropometric measurements for the top three principle components (PC), which explained 53% of the results. PC2 vs PC1 (A), PC3 vs PC1 (B), and PC3 vs PC2 (C).

Scatter plots of PC2 vs. PC1, PC3 vs. PC1, PC3 vs. PC2 are shown in Fig. 2A, B, and 2C, respectively. From Fig. 2A *Nilotic* (Kotido) women seem to be distinctively separate from the *Bantu* (Kiboga, Ngoma, and Mbarara) women. Kotido women tend to be high with respect to PC2 but low with respect to PC1, which may indicate they tend to be taller/longer women but with less girth and weight. Also, scatter plots in Fig. 2B and C, the co-clustering of *Nilotic* with *Bantu* is minimal. Does location matter within the *Bantu*? By examining Fig. 2B and C, the answer is no as the co-clustering of Kiboga and Mbarara is evident. However, Fig. 2A reveals a slightly different story – Kiboga spreads out along PC1 axis and clusters less with the other Bantu locations. Kiboga and Mbarara women tend to be high with respect to PC1, which may indicate that they tend to be larger in terms of size and weight. It should be pointed out that these observations are preliminary; however, they are important in providing insights in hypotheses to be tested with more data. PCA generally requires a large number of observations in order to be unequivocally conclusive. One rule of thumb is that a data set should contain 10 observations for each variable in the PCA (Hair et al., 1998). The fact that we have 28 variables and only 89 observations, a case can be made for further measurements. Generally, our study is large enough to conclusively illustrate differences between *Bantu* and *Nilotic*, but possibly not large enough to rule out or confirm differences among *Bantu,* based on location.

To gain better insights in the PCA results, we further determined which measurements in general were different among the groups. For each measurement, a different ANOVA was conducted to compare the women from different locations. The F statistic, degree of freedom and p-values are provided in Table 3. Eighteen out of the 28 returned a p-values less than 0.05 and in agreement with PCA, in each of these measurements Kotido is either less or greater than one or a combination of the other measurements. Among the *Bantu* groups Kiboga seems to most frequently differ from the others. This is in contrast to our expectations and we have no explanation to offer at this time.

Stature is a very important anthropometric measurement as it is used in equations or models to predict other dimensions (Agrawal et al., 2010). The statures for the groups were not significantly different (Table 3). Since there are differences not only between the *Bantu* and *Nilotic*, but also among the *Bantu* (Kiboga versus the other) (Table 3), there is a possibility that different prediction equations will be needed for different groups even among the same ethnolinguistic group. A large database is therefore required to determine which groups cluster together and for whom a single applicable equation can be developed.

### 3.2 Comparison of actual and estimated mesuremets

To indirectly compare the Ugandan to the American women, we assessed how well predictive models based on American women anthropometric data estimate Ugandan women measurements. Table 4 shows the three equations used and the resulting prediction errors. We can see that eye height-standing, eye height-seated are both underestimated by the model. Of the two models, the difference is more pronounced with the eye height-seated model. But in both cases the errors are considered reasonable. The Acromion height is grossly overestimated by the model. The source of this error is at least from sitting height since the difference between the Ugandan and American women is significantly high as shown in the Supplementary Table 1. Waist circumference for American women was not available for direct comparison. The take-home message is that some measurements between the Ugandan and American women are identical, but there are some that are not. It is important to know which measurements are identical and which are not, so new prediction models can be established. Since differences have been observed with Ugandan women, it is highly likely that differences will be observed between American and other sub-Saharan African women. Also if a wider net is cast over diverse sub-Saharan women covering many ethnic groups, differences are expected along not only ethnic lines, but also along geographical location within the same ethnic group. Differences in local nutrition will likely be the major factor (Chandra et al., 2013, Chandra et al., 2011). Knowledge of these differences is important in creating practical prediction models.

Anthropometric data are typically presented in percentile form to inform design. The 5th percentile values are used for design where the lower limit is the restrictive factor such as sitting height, stature, crank handle-length and grip-diameter. The 95th percentile is used in design where the upper limit is the restrictive factor such as grip-length. A comparison of Ugandan women groups and American women percentile ranges are presented in Supplementary Table 1. As expected, in some cases the range is close and in others it is not. We illustrate the importance of these differences with respect to informing our design of the milk churner described in detail elsewhere (Kisaalita et al., 2015; see supplementary video clip attached).

Supplementary video related to this article can be found at http://dx.doi.org/10.1016/j.apergo.2015.12.010.

The following is the supplementary data related to this article:

Supplementary Video A Nilotic woman churning fermented milk in a hand-operated churner design informed by anthropometric measurements.2

### 3.3 Milk churner design informed by anthropometry

In our observations of the traditional churning process in gourds, which are containers made from large dried fruit of the plant *Lagenera peucantha,* and discussions with women churners (Kisaalita et al., 2015; also see video clips associated with this reference), two characteristics turned-up as critical to new churner design usability. The two characteristics were: churner height (preference of operation while seated as opposed to standing) and crank dimensions (need for reduction of the energy required for the separation/gourd shaking and resultant long-term pain in the hand, arm and chest). We use the relevant anthropometric measurements, such as arm span and grip diameter-inside, to further make the case for establishing stand-alone anthropometry database for sub-Saharan African women.

#### 3.3.1 Milk churner height

**Fig. 3:**
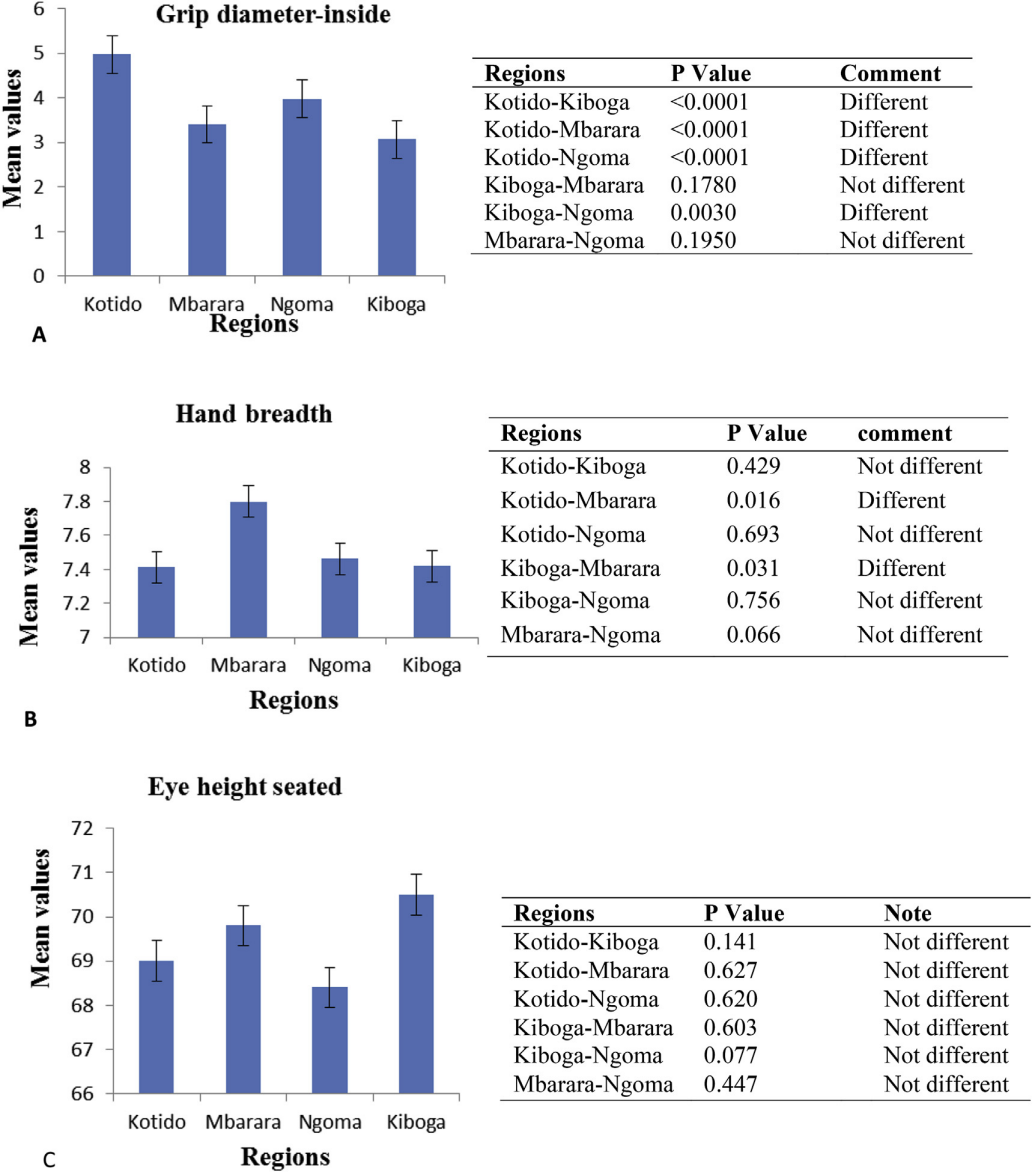
Pairwise comparison of women groups for eye height-seated (A), grip diameter-inside (B), and hand breadth (C). Side tables show the t-test p-values.

Eye height-seated is a very important dimension as it is pertinent to the machine user's positioning in order to watch the machine output. From the bar graph (Fig. 3A), Kiboga revealed the highest value, followed by Mbarara, Kotido, and lastly Ngoma. Women who are bigger tend to have a raised buttocks height, which tends to affect the eye height-seated. This has been observed with two individuals of the same stature but with different weights on the same seat, the well-built individual exhibited a raised body while seated. This would explain Kiboga's highest value. However, the paired t-test results (Fig. 3A side table) showed no significant differences among the groups (p < 0.05) for eye height-seated.

Designers have used the eye height-seated to size height of operating tools (Deros et al., 2009); however, no standard applicable calculation to size the churner height was found. We sized the churner height (floor to connection) as equal to the sum of 5th percentile values of elbow height, popliteal height and crank arm length. The calculated values are shown in Table 5. The American women value is 4 cm lower than the highest Kotido value, which is significant. In absence of Ugandan women anthropometric data, used of American women data would have resulted in an undersized churner with respect to height. Whether the value we selected of 86 cm is the “best” for the Ugandan women is a question to be answered in churner usability studies.

#### 3.3.2 Crank grip diameter and grip length

Grip diameter affects the grip strength through the biomechanics of grip and Radwin and Haney (1996) has recommended a diameter of 3.8 cm, which incidentally is the 5th percentile value for American women grip diameter-inside (see Supplementary Table 1). Kotido women 5th percentile grip diameter-inside was found to be 4.2 cm, larger than that for American women. However, for the rest of the Ugandan women, a much smaller value of 2.1 cm was found. Again, while use of the American women database would have served well for Kotido, it would have oversized the grip diameter for the other Ugandan women. The t-test analysis results reported in Fig. 3B also show Kotido women grip diameter-inside to be higher than the rest of the women, underscoring the difference between *Bantu* and *Nilotic* women. In this case there were no significant differences based on *Bantu* women location. To size the churner crank grip diameter, we followed Singh et al. (2013) recommended calculation of 130% of the lowest grip diameter-inside of 2.1 cm yielding a design value of 2.73 cm [1.3 × 2.1 cm]. Interestingly, the minimum grip diameter was found by Agrawal et al. (2010) to be 3.3 cm for female Indian farm workers. A case study of farm hand-tools injuries in northern India revealed that the mechanism of injuries was slippage of the tool (Kumar et al., 2008). Intuitively, an oversized grip diameter is more prone to slipping out of the hand of the operator.

According to Singh et al. (2013), the design of crank grip length should be based on 95% percentile measurements of hand breadth. As shown in Fig. 3C, Mbarara women's hand breadth was significantly larger than the rest of the women groups, but lower than the American women's. We sized the crank grip length following Radwin and Haney (1996) recommendation of 130% of the largest 95% percentile hand breadth, which yielded a value of 10.8 cm (1.3 × 8.3 cm). Again, the design value for Ugandan women is smaller than that for American women by approximately 0.5 cm. The minimum crank grip length for Indian female workers was found to be 9.5 cm (Singh et al., 2013). However, Radwin and Haney (1996) in their study noted that the recommended grip length is in the range of 6.4–8.9 cm to achieve high grip forces.

#### 3.3.3 Crank arm length

According to Agrawal et al. (2010), the crank arm holding height is dependent of the elbow height and permitted range of elbow angle. The comfortable range of angle in this case for the churner operation should at least be in the range of 90–100^0^. Typical crank arm length for hand-tools is 25.0 cm and apparently there is no scientific rationale for this setting (Neville et al., 2010). Studies for best crank arm length with respect to torque output for women in Indian and United Kingdom are in the range of 17.5–30.0 cm (Neville et al., 2010, Singh et al., 2013). An optimum crank arm length of 27.0 cm was found most appropriate for optimum torque (Singh et al., 2013).

In standing arm-crank ergonomics, Neville et al. (2010) found the optimal crank arm length for maximum power to be 12–12.5% of the arm span. We had not included arm span in our original list of anthropometric measurement. We extended our studies by fitting the milk churner with adjustable crank arm lengths of 17.5, 20.0, 27.0 and 30.0 cm. With no load (i.e., no milk in the churner), we asked women at Ngoma (6) and Kiboga (16) to crank the churner and decide the best crank arm length. We also measured these participants’ arm span (the results are reported in Supplementary Table 1). Our expectations were that the women will show a preference of approximately 12.5 of their arm span, which is the 20.0 cm-length. Out of the 23 women tested, seven selected the 17.5 cm-length, five elected the 20 cm-length, eight selected the 27 cm-length, and two selected 30 cm-length. There was no discernible relationship between the arm span length and the selected crank arm length. Detailed studies for the optimum crank arm length under different load composition are needed to inform future hand-tool design. We noticed that the stature of all these women were approximately equal to the arm span length. So we used the stature for groups of women, for whom arm span length was not available, to calculate crank arm lengths presented in Table 5.

## 4 Conclusion

*Bantu* and *Nilotic* women of Uganda are different with respect to anthropometric measurements. But differences within *Bantu* women based on location are inconclusive. Overall, models based on American women anthropometric data do not adequately predict body dimensions for Ugandan women and probably other sub-Saharan African women. More data collection is need for more robust statistical hypothesis testing of what measurements are universally equal or different and for development of models that more accurately predict body dimensions using easily measurable dimensions as independent variables. These models will go a long way in facilitating the development of better hand-tools for the underserved sub-Saharan women.

## Figures and Tables

**Table 1:** Land marks and body dimensions measured.

No.	Landmarks	Measurement taken (see Fig. 1)
	**Direct**	
1.	Acromiale	Arm length (01)
2.	Radiale	Fore arm length (02)
3.	Stylion	Hand length (03)
4.	Techontarion	Thigh length (04)
5.	Tibiale laterale	Tibiale laterale (knee) height (05)
6.	Tibiale mediale	Tibiale mediale–sphyrion tibiale (leg) length (NS^a^)
7.		Arm reach from the wall (06)
8.	Sphyrion tibiale	Foot length (07)
9.		Stature while standing (08)
10.	**Indirect**	Weight (09)
11.	Middle cromiale-radiale	Arm girth when relaxed (NS)
12.		Arm girth when flexed and tensed (NS)
13.	Mid Stylion	Wrist circumference (10)
14.		Acromial height when sitted (11)
15.		Eye height when sitted (12)
16.		Biepicondular humus breadth (elbow girth) (NS)
17.		Bistyloid wrist breadth (NS)
18.		Hand breadth (13)
19.		Popliteal height (14)
20.		Elbow rest height (15)
21.		Coronoid fossa to hand length (16)
22.		Tricipetal skinfold thickness (NS)
23.		Grip diameter (inside) (17)
24.		Grip diameter (outside) (18)
25.		Calf circumference (19)
26.		Sitting height (20)
27.		Eye height when standing (21)
28.		Waist circumference (22)

a NS = Not shown in Fig. 1.

**Table 2:** Participants’ distribution.

Large ethnolinguistic grouping	Tribe/Location	Number of participants	Percentage
Bantu	Bahima/Kiboga	27	30.3%
	Bahima/Ngoma	21	23.6%
	Bahima-Bahororo/Mbarara	9	10.1
	Bantu Total	57	**64%**
Nilotic	Jie/Kotido	32	
	Nilotic Total	32	**36%**
Total		89	100%

**Table 3:** Comparison of measurements across locations.

Measure	F-Value	p-Value	Comment
Arm length	3.98	**0.0105**	Kiboga > Kotido
Forearm length	1.00	0.3987	
Hand length	1.29	0.2835	
Thigh length	3.16	**0.0289**	Kiboga > Ngoma
Knee height	3.01	0.0345	No difference after Tukey adjustment
Leg length	4.70	**0.0044**	Mbarara > Kotido
Arm reach	1.98	0.1231	
Foot length	1.24	0.3020	
Stature	1.95	0.1283	
Weight	39.13	**<0.0001**	All others > Kotido
Arm girth relaxed	26.36	**<0.0001**	All others > Kotido
Arm girth flexed	22.89	**<0.0001**	All others > Kotido
Wrist circumference	4.66	**0.0046**	Mbarara > All others
Acromial height seated	3.42	**0.0208**	Kiboga > Ngoma
Eye height seated	1.33	0.2705	
Elbow girth	4.67	**0.0045**	Kotido > Kiboga
Wrist breadth	23.29	**<0.0001**	Mbarara > Ngoma, Kotido; Kiboga > Kotido
Hand breadth	1.87	0.1414	
Popliteal height	4.12	**0.0089**	Kotido > Kiboga, Ngoma
Elbow rest height	7.13	**0.0002**	Kiboga > Kotido, Ngoma
Coronoid fossa to hand length	2.52	0.0636	
Tricipital skinfold thickness	73.14	**<0.0001**	All others > Kotido
Grip diameter inside	35.96	**<0.0001**	All others > Kotido
Grip diameter outside	6.65	**0.0004**	Mbarara > Kotido
Calf circumference	16.86	**<0.0001**	All others > Kotido
Waist circumference	22.46	**<0.0001**	All others > Kotido
Sitting height	1.98	0.1235	
Eye height standing	0.83	0.4790	

Statistical significance – p-vales in bold are less than 0.05.

**Table 4:** Prediction equations and prediction error.

Parameter	Models (Kroemer et al., 1997)	Prediction error^a^ (%)	
Direct values	Absolute values
Eye height standing	= [(0.963 × Stature while standing (cm))−5.7101]	Kotido = 0.52	1.12
Kiboga = 0.73	1.23
Mbarara = 0.57	0.67
Ngoma = 1.28	2.06
All = 0.78	1.33
Eye height seated	= [(0.907 × Sitting height (cm))− 3.7877]	Kotido = −7.85	14.10
Kiboga = 4.72	4.72
Mbarara = 3.06	3.16
Ngoma = 4.28	4.58
All = 0.07	7.90
Acromion Height	= [(0.957 × Stature while standing (cm))−(0.208 × Sitting height (cm))+(0.065 × Waist circumference (cm))−(9.6449)]	Kotido = −42.96	42.96
Kiboga = −39.06	39.06
Mbarara = −41.92	41.92
Ngoma = −42.57	42.57
All = −41.57	41.57

a Prediction error = [(measure value – estimate value)/measured value)] *x*100.

**Table 5:** Informing milk churner design by Ugandan women anthropometry (all dimensions in cm).

Design parameter	Design approach	Kotido	Kiboga	Mbarara	Ngoma	American
Churner height	Machine Height = Elbow Height + Popliteal height + Crank Length. Values at 5th percentile.	86.8	86.6	85.6	84.8	82.7
Churner crank arm grip diameter	5^th^ percentile of the inside grip diameter	4.2	2.1	2.1	2.1	3.8
Churner crank arm grip length	95% hand breadth + (30% of 95% hand breadth)	10.5	10.6	10.8	10.7	11.2
Churner crank arm length	Crank length is 12.5% of the mean Arm Span. Arm Span is directly proportional to the Stature. Therefore from data Crank Length was taken as 12.5% of stature	21.0	20.5	20.7	20.9	20.4
