# Supplementary figures and images for: Anthropometric characteristics of female smallholder farmers of Uganda – Toward design of labor-saving tools
Source: Appl Ergon. 2016 May;54:177–85. doi: 10.1016/j.apergo.2015.12.010 (PMC4754207; doi:10.1016/j.apergo.2015.12.010)

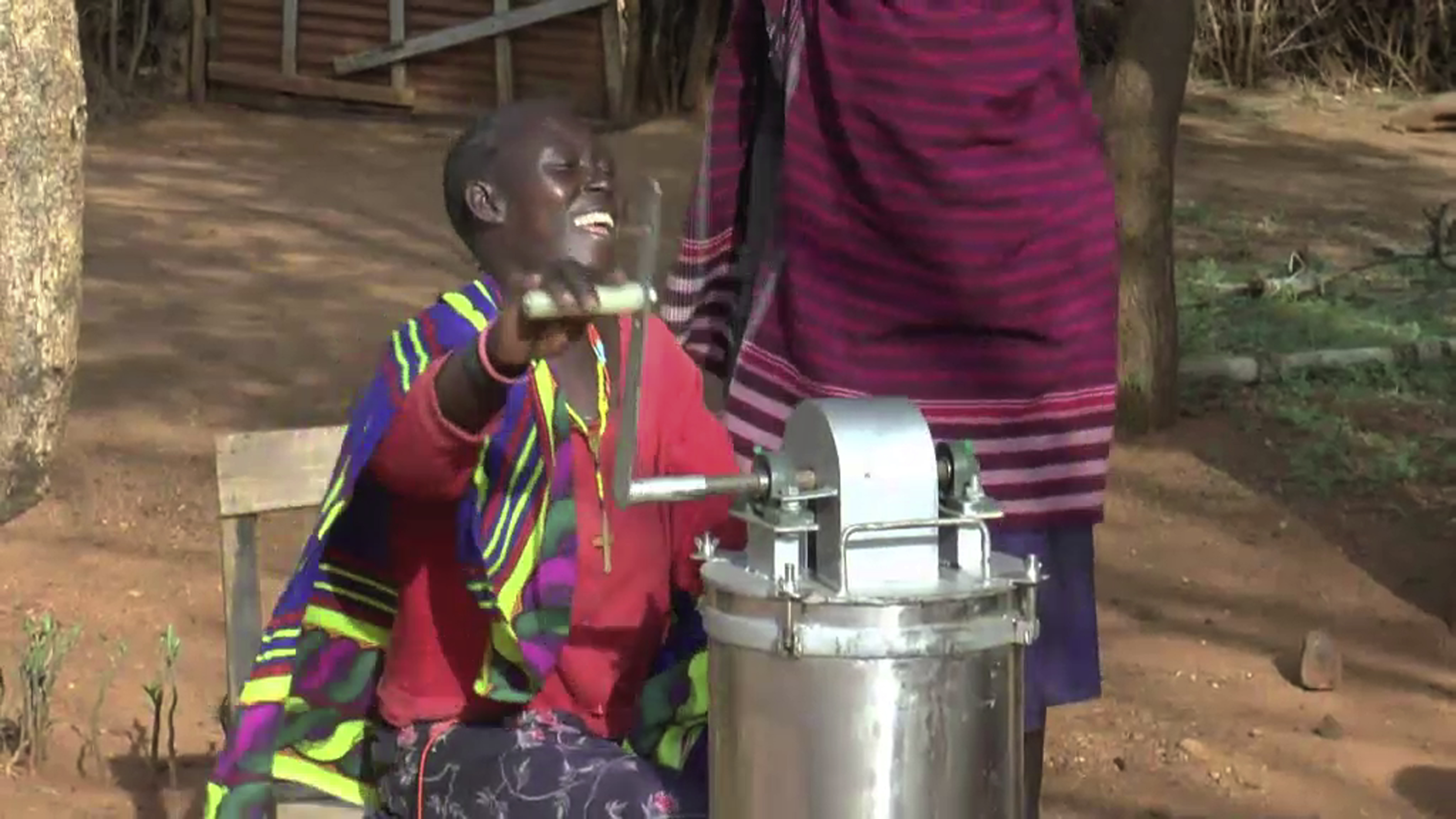

Supplement: Supplementary Video — A Nilotic woman churning fermented milk in a hand-operated churner design informed by anthropometric measurements.2 [file mmc2.jpg]
