# Supplementary material for: Anthropometric characteristics of female smallholder farmers of Uganda – Toward design of labor-saving tools
Source: Appl Ergon. 2016 May;54:177–85. doi: 10.1016/j.apergo.2015.12.010 (PMC4754207; doi:10.1016/j.apergo.2015.12.010)
Supplement: Supplementary file 2 [file mmc1.doc]

Supplementary Table 1.Comparison of Ugandan measured women to American women anthropometric values

| **No.** | Measurements (units) | Regions | | | | | | | | | | | | | | | | | | | |
| --- | --- | --- | --- | --- | --- | --- | --- | --- | --- | --- | --- | --- | --- | --- | --- | --- | --- | --- | --- | --- | --- |
|  |  | Kotido | | | | Kiboga | | | | Mbarara | | | | Ngoma | | | | America[[1]](#endnote-2) | | | |
|  |  | std | Mean | 5th | 95th | std | Mean | 5th | 95th | std | Mean | 5th | 95th | std | Mean | 5th | 95th | std[[2]](#endnote-3) | Mean | 5th | 95th |
| **1** | Arm length (cm) | 1.8 | 32.4 | 29.5 | 35.3 | 2.0 | 34.1 | 30.8 | 37.3 | 3.2 | 32.7 | 27.4 | 38.1 | 2.4 | 33.5 | 29.6 | 37.4 |  |  |  |  |
| **2** | Fore arm length (cm) | 2.0 | 26.1 | 22.8 | 29.4 | 1.6 | 26.0 | 23.4 | 28.6 | 1.1 | 26.7 | 24.9 | 28.5 | 1.7 | 25.6 | 22.8 | 28.3 |  |  |  |  |
| **3** | Handle length (cm) | 1.1 | 19.4 | 17.6 | 21.2 | 0.9 | 19.0 | 17.5 | 20.4 | 1.2 | 19.4 | 17.3 | 21.4 | 1.2 | 18.9 | 17.0 | 20.8 | 1.0 | 18.1 | 16.5 | 19.7 |
| **4** | Thigh length (cm) | 3.0 | 42.4 | 37.5 | 47.3 | 2.9 | 40.4 | 35.7 | 45.1 | 4.5 | 40.0 | 32.7 | 47.4 | 2.7 | 40.2 | 35.8 | 44.5 |  |  |  |  |
| **5** | Knee height (cm) | 2.6 | 47.3 | 43.1 | 51.5 | 3.1 | 48.1 | 43.0 | 53.2 | 1.5 | 49.5 | 47.1 | 52.0 | 2.3 | 48.9 | 45.0 | 52.7 |  |  |  |  |
| **6** | Leg length (cm) | 3.3 | 38.4 | 33.0 | 43.8 | 8.2 | 39.1 | 25.7 | 52.5 | 1.2 | 41.0 | 39.1 | 42.9 | 2.0 | 40.3 | 37.0 | 43.7 | - | 57.4 | 53.1 | 61.7 |
| **7** | Arm reach (cm) | 17.6 | 77.5 | 48.6 | 106.3 | 5.6 | 74.3 | 65.2 | 83.4 | 5.2 | 75.1 | 66.5 | 83.6 | 3.3 | 72.6 | 67.2 | 77.9 |  |  |  |  |
| **8** | Foot length (cm) | 1.1 | 24.8 | 23.0 | 26.7 | 1.1 | 24.2 | 22.5 | 26.0 | 1.5 | 24.6 | 22.2 | 27.0 | 0.9 | 24.5 | 23.0 | 25.9 | 1.2 | 24.4 | 22.4 | 26.5 |
| **9** | Stature (cm) | 5.2 | 167.4 | 158.9 | 175.9 | 5.3 | 166.4 | 157.7 | 175.0 | 5.9 | 168.0 | 158.4 | 177.6 | 6.5 | 163.7 | 153.0 | 174.3 | 6.4 | 162.9 | 152.8 | 173.7 |
| **10** | Weight (kg) | 16.4 | 60.8 | 33.9 | 87.6 | 15.8 | 93.6 | 67.7 | 119.5 | 12.9 | 92.0 | 70.9 | 113.1 | 16.1 | 84.3 | 57.8 | 110.7 | 14.0 | 62.0 | 39.0 | 85.0 |
| **11** | Arm girth-relaxed (cm) | 2.6 | 25.9 | 21.6 | 30.2 | 6.4 | 37.0 | 26.5 | 47.5 | 6.7 | 36.9 | 25.9 | 48.0 | 6.3 | 36.3 | 26.0 | 46.6 |  |  |  |  |
| **12** | Arm girth- flexed & tensed (cm) | 2.7 | 26.7 | 22.3 | 31.1 | 6.4 | 37.0 | 26.5 | 47.5 | 6.7 | 36.9 | 26.0 | 47.9 | 6.3 | 36.4 | 26.1 | 46.7 |  |  |  |  |
| **13** | Wrist circumference (cm) | 0.7 | 15.6 | 14.4 | 16.8 | 0.8 | 15.7 | 14.3 | 17.1 | 1.2 | 16.7 | 14.7 | 18.7 | 30.9 | 22.1 | -28.7 | 72.8 |  |  |  |  |
| **14** | Acromial height (cm) | 2.9 | 92.1 | 87.3 | 96.8 | 12.7 | 92.0 | 71.1 | 112.8 | 2.8 | 92.9 | 88.4 | 97.5 | 6.3 | 90.8 | 80.5 | 101.1 |  |  |  |  |
| **15** | Eye height-seated (cm) | 4.0 | 69.0 | 62.5 | 75.6 | 3.4 | 70.5 | 65.0 | 76.0 | 3.8 | 69.8 | 63.6 | 76.0 | 4.3 | 68.4 | 61.3 | 75.6 | 3.3 | 73.9 | 68.5 | 79.4 |
| **16** | Elbow girth (cm) | 0.4 | 5.9 | 5.2 | 6.6 | 0.5 | 5.4 | 4.6 | 6.1 | 0.7 | 5.6 | 4.5 | 6.8 | 0.5 | 5.6 | 4.7 | 6.5 |  |  |  |  |
| **17** | Wrist breadth (cm) | 2.2 | 4.9 | 1.2 | 8.6 | 0.4 | 5.1 | 4.5 | 5.7 | 0.3 | 5.3 | 4.8 | 5.7 | 2.0 | 5.3 | 2.1 | 8.5 |  |  |  |  |
| **18** | Hand breadth (cm) | 0.4 | 7.4 | 6.7 | 8.1 | 0.5 | 7.4 | 6.7 | 8.2 | 0.3 | 7.8 | 7.3 | 8.3 | 0.5 | 7.5 | 6.7 | 8.2 | 0.4 | 7.3 | 7.9 | 8.6 |
| **19** | Popliteal height (cm) | 2.1 | 44.2 | 40.8 | 47.6 | 2.3 | 42.3 | 38.5 | 46.1 | 1.7 | 43.2 | 40.4 | 46.0 | 2.3 | 42.4 | 38.6 | 46.2 | 2.4 | 35.1 | 38.9 | 42.9 |
| **20** | Elbow rest height (cm) | 2.5 | 20.2 | 16.1 | 24.3 | 2.9 | 23.4 | 18.6 | 28.2 | 4.0 | 21.7 | 15.2 | 28.3 | 2.6 | 20.4 | 16.1 | 24.7 |  |  |  |  |
| **21** | Coronoid fossa-hand length (cm) | 2.4 | 45.5 | 41.6 | 49.4 | 1.9 | 45.8 | 42.7 | 48.8 | 2.1 | 45.3 | 41.9 | 48.8 | 2.7 | 44.0 | 39.6 | 48.3 |  |  |  |  |
| **22** | Skinfold thickness (mm) | 6.5 | 24,2 | 13.6 | 34.8 | 11.8 | 59.4 | 40.0 | 78.9 | 8.0 | 56.1 | 42.9 | 69.3 | 18.2 | 61.0 | 31.0 | 90.9 |  |  |  |  |
| **23** | Grip diameter(inside) (cm) | 0.4 | 5.0 | 4.2 | 5.7 | 0.6 | 3.1 | 2.1 | 4.0 | 0.8 | 3.4 | 2.1 | 4.7 | 1.1 | 4.0 | 2.1 | 5.8 | - | 4.3 | 3.8 | 4.8 |
| **24** | Grip diameter(outside) (cm) | 0.5 | 6.8 | 6.0 | 7.6 | 0.6 | 7.4 | 6.4 | 8.4 | 0.7 | 7.4 | 6.3 | 8.5 | 0.4 | 7.1 | 6.5 | 7.8 |  |  |  |  |
| **25** | Calf circumference (cm) | 2.4 | 31.8 | 27.9 | 35.8 | 4.4 | 36.5 | 29.3 | 43.6 | 5.1 | 37.2 | 28.8 | 45.6 | 4.2 | 39.2 | 32.3 | 46.0 |  |  |  |  |
| **26** | Waist circumference (cm) | 5.9 | 77.1 | 67.4 | 86.8 | 12.2 | 95.1 | 75.2 | 115.1 | 19.3 | 91.1 | 59.4 | 122.8 | 11.9 | 95.4 | 75.9 | 114.8 |  |  |  |  |
| **27** | Sitting height (cm) | 6.0 | 118.6 | 108.8 | 128.4 | 4.1 | 118.7 | 112.0 | 125.4 | 4.2 | 119.9 | 113.0 | 126.7 | 4.1 | 117.0 | 110.2 | 123.8 | 3.5 | 85.2 | 79.5 | 91.0 |
| **28** | Eye height-standing (cm) | 5.6 | 156.4 | 147.1 | 165.6 | 5.2 | 155.7 | 147.1 | 164.3 | 6.4 | 157.0 | 146.4 | 167.6 | 5.7 | 153.9 | 144.6 | 163.3 | 6.3 | 151.6 | 151.6 | 162.1 |
| **29** | Span (cm)[[3]](#endnote-4) |  |  |  |  | 6.2 | 174.4 | 164.3 | 184.5 |  |  |  |  | 3.1 | 171.7 | 166.6 | 176.8 | 8.1 | 167.2 | 154.2 | 180.9 |

1. Values obtained from Gordon, C.C.; Churchill, T.; Clauser, C.E.; Bradtmiller, B.; McConvile, J.T.; Teddetts, I.; Walker, R.A. 1988. Anthropometric Survey of US Army Personnel: Summary Statistics Interim Report. Natick TR-89/027. Natick, MA: US Army Natick Research, Development and Engineering Center. [↑](#endnote-ref-2)
2. Values presented without a standard deviation were obtained from Kodak’s Ergonomic Design for People at Work, 2nf Ed., Table 1.5, pp 48-49. [↑](#endnote-ref-3)
3. The values presented were measured later with fewer numbers of participants; Kiboga, n=6; Ngoma, n=12. [↑](#endnote-ref-4)
